# Supplementary material for: Sida Golden Mosaic Virus, an Emerging Pathogen of Snap Bean (Phaseolus vulgaris L.) in the Southeastern United States
Source: Viruses. 2023 Jan 26;15(2):357. doi: 10.3390/v15020357 (PMC9959804; doi:10.3390/v15020357)
Supplement: Supplementary file 1 [file viruses-15-00357-s001.zip › viruses-2158889-supplementary.pdf]

viruses

MDPI

Article

Sida golden mosaic virus, an emerging pathogen of snap bean (*Phaseolus vulgaris* L.) in the southeastern United States

Saurabh Gautam [1], James W. Buck [2], Bhabesh Dutta [3], Timothy Coolong [4], Tatiana Sanchez[5], Hugh A. Smith[6], Scott Adkins[5] and Rajagopalbabu Srinivasan[1]*

[1] Department of Entomology, University of Georgia, 1109 Experiment Street, Griffin, GA 30223, USA
[2] Department of Plant Pathology, University of Georgia, 1109 Experiment St., Griffin, GA 30223, USA
[3] Department of Plant Pathology, University of Georgia, 3250 Rainwater Road, Tifton, GA 31793, USA
[4] Department of Horticulture, University of Georgia, 1111 Miller Plant Sciences, Athens, GA, USA
[5] University of Florida, IFAS Extension, 22712 W. Newberry Road, Newberry, FL 32669, USA
[6] Department of Entomology and Nematology, University of Florida, 14625 Co Rd 672, Wimauma, FL 33598, USA
[7] USDA-ARS, U.S. Horticultural Research Laboratory, Fort Pierce, FL 34945, USA
* Correspondence: babusri@uga.edu

S.1 Surface sterilization of plants and insects

The youngest leaf of the test plants (100mg) or insects were collected and surface sterilized by washing with 1% bleach for 30 sec, then rinsing with autoclaved distilled water, then by a single wash with 75% ethanol for 30 sec, and finally three rinses with autoclaved distilled water. Water from the last rinse was collected in a 1.5 ml autoclaved centrifuge tube. Using the GeneJET Plant Genomic DNA Purification Kit, the total DNA from surface-sterilized tissues was extracted. Water from the last rinse (4 µl) and extracted total genomic DNA (20 ng) was subjected to PCR analysis using primers SiGMVF and SiGMVR as described Below. Surface sterilization and PCR of water from the last rinse ensured elimination of false positives that may arise due to residual honeydew present on the leaf surface.

Table S1. Primers used for amplification of a segment of DNA-A of SiGMV.

	Name	Sequence (5′ – 3′)	Product size (bp)
Endpoint PCR	SiGMVF	TTCTCCTCGTGCAGGTAGTG	574
	SiGMVR	ACTTGCCAGCCTCTTGATGA	
QPCR	SiGMV-QF	CTCAAAGGTTAGCCGCAACG	114
	SiGMV-QR	CGGTAGATCCTGGGCTTCCT	

SiGMV- sida golden mosaic virus.

Table S2. Sida viruses used for phylogenetic analysis.

SN	Viruses	GenBank (Access. No.)	Acronym	Location [b]
1	Sida angular mosaic virus	KX691407	SiAMV	BR (NW)
2	Sida bright yellow mosaic virus	KX348184	SiBYMV	BR (NW)
3	Sida chlorotic mottle virus	KX348183	SiCMoV	BR (NW)
4	Sida chlorotic vein virus	KX691402	SiCVV	BR (NW)
5	Sida ciliaris golden mosaic virus	JX857691	SicGMV	VE (NW)
6	Sida common mosaic virus	EU710751	SiCMV	BR (NW)
7	Sida golden mosaic Braco virus-[Jamaica:Liguanea:2008]	NC_038458	SiGMBcV	JM (NW)
8	Sida golden mosaic Brazil virus	FN436001	SiGMBRV	BR(NW)
9	Sida golden mosaic Buckup virus-[Jamaica:St. Elizabeth:2004]	NC_014794	SiGMBuV	JM (NW
10	Sida golden mosaic Costa Rica virus	NC_004657	SiGMCRV	CR (NW)
11	Sida golden mosaic Florida virus-Malvastrum	NC_014446	SiGMFV-Malvastrum	USA(NW)
12	Sida golden mosaic Florida virus-USA	U77963	SiGMFV-A1	USA(NW)
13	Sida golden mosaic Honduras virus	NC_004659	SiGMHV	HN (NW)
14	Sida golden mosaic Lara virus	NC_038459	SiGMLaV	VE (NW)
15	Sida golden mosaic Liguanea virus-[Jamaica:1:2008]	HQ009522	SiGMLigVJM	JM(NW)
16	Sida golden mosaic virus - Florida [Phaseolus]	GQ357649	SiGMV	USA(NW)
17	Sida golden mosaic virus - whitefly VEM Citra Florida	HM626517	SiGMV-[US:FlL:Cit:07:VEM]	USA(NW)
18	Sida golden mottle virus	NC_014130	SiGMoV	USA(NW)
19	Sida golden yellow spot virus	NC_038992	SiGYSV	BR(NW)
20	Sida golden yellow vein virus-[A11]	NC_038460	SiGYVV-[US:Hom:A11]	USA(NW)
21	Sida golden yellow vein virus-[Jamaica:Liguanea2:2008]	NC_004635	SiGYVV-[JM:Lig2:08]	JM(NW)
22	Sida golden yellow vein virus-Malvastrum	JN411689	SiGYVV-Malvastrum	CU(NW)
23	Sida golden yellow vein virus-Malvastrum [CU:Camaguey 177:09]	HQ896203	SiGYVV-Malvastrum-CU	CU(NW)

24	Sida leaf curl virus	NC_007638	SiLCuV	CN(OW)
25	Sida micrantha mosaic virus - [Brazil:okra]	EU908733	SiMMV-[Br:Ok]	BR(NW)
26	Sida micrantha mosaic virus - Rhombifolia [Bolivia:Boyuibe-Villamontes:2007]	HM585431	SiMMV/Rho-[Bolivia:Boyuibe-Villamontes]	BL(NW)
27	Sida micrantha mosaic virus - Rhombifolia [Bolivia:Cerro Fraile 1:2007]	HM585437	SiMMV/Rho-[Bolivia:Cerro Fraile_1]	BL(NW)
28	Sida micrantha mosaic virus - Rhombifolia [Bolivia:Cerro Fraile 2:2007]	HM585439	SiMMV/Rho	BL(NW)
29	Sida micrantha mosaic virus [Brazil:Mato grosso do sul:2:2007] - [Bolivia]	HM585433	SiMMV-[Br:Mato grosso do sul:2]	BR(NW)
30	Sida micrantha mosaic virus-[snap bean:Brazil:2003]	HM357459	SiMMV-[Br: SB]	BR(NW)
31	Sida mosaic Alagoas virus	NC_016573	SiMALV	BR(NW)
32	Sida mosaic Bolivia virus 1	NC_015046	SiMBoV1	BL(NW)
33	Sida mosaic Bolivia virus 2	NC_015043	SiMBV2	BL(NW)
34	Sida mosaic Sinaloa virus	NC_008059	SiMSiV	MX(NW)
35	Sida mottle Alagoas virus	NC_020256	SiMoAV	BR(NW)
36	Sida mottle virus-[Brazil]	AY090555	SiMoV/Rho	BR(NW)
37	Sida yellow blotch virus	NC_020254	SiYBV	BR(NW)
38	Sida yellow leaf curl virus	NC_038461	SiYLCV	BR(NW)
39	Sida yellow mosaic Alagoas virus	NC_020255	SiYMAV	BR(NW)
40	Sida yellow mosaic China virus - [Hainan 8]	NC_017987	SiYMCNV	CN(OW)
41	Sida yellow mosaic virus-[Brazil]	NC_004639	SiYMV	BR(NW)
42	Sida yellow mosaic Yucatan virus	NC_008779	SiYMYuV	MX(NW)
43	Sida yellow mottle virus [Cuba:Sancti Spiritus 159:2009]	HQ822123	SiYMoV	CU(NW)
44	Sida yellow net virus	NC_020253	SiYNV	BR(NW)
45	Sida yellow vein Madurai virus	NC_009354	SiYVMV	IN(OW)
46	Sida yellow vein Vietnam virus	NC_009547	SiYVVV	VN(OW)
47	Sida golden mosaic virus	MK387701	SiGMV[a]	USA (NW)

[a]SiGMV sequence obtained in this study. [b] Locations: NW (New World), OW (Old World), HN (Honduras), VN (Vietnam), IN (India), BR (Brazil), CU (Cuba), MX (Mexico), CN (China), VE (Venezuela), CR (Costa Rica), JM (Jamaica), BL (Bolivia), USA (United States of America).
